# Hyperinflation deteriorates arterial oxygenation and lung injury in a rabbit model of ARDS with repeated open endotracheal suctioning

**DOI:** 10.1186/s12871-015-0045-5

**Published:** 2015-05-06

**Authors:** Junko Kamiyama, Subrina Jesmin, Hideaki Sakuramoto, Nobutake Shimojyo, Majedul Islam, Keiichi Hagiya, Masato Sugano, Takeshi Unoki, Masami Oki, Satoru Kawano, Taro Mizutani

**Affiliations:** Department of Emergency and Critical Care Medicine, Faculty of Medicine, University of Tsukuba, Ibaraki, Japan

**Keywords:** ARDS, Open endotracheal suctioning, Hyperinflation, Arterial oxygenation, TNF-α, IL-8

## Abstract

**Background:**

Hyperinflation (HI) is performed following open endotracheal suctioning (OES), whose goals include: to stimulate a cough, recover oxygenation and improve compliance. However, it may also induce unintended consequences, including: lung stress and strain, failure to maintain high distending pressure, and subsequently cycling recruitment and derecruitment. Here, our aim was to investigate the effects of hyperinflation after repeated OES on sequential alteration of arterial oxygenation and lung injury profile using a saline lavage-induced surfactant depleted ARDS rabbit model.

**Methods:**

Briefly, 30 Japanese White Rabbits were anesthetized and ventilated in pressure-controlled setting with a tidal volume of 6-8 ml/kg. Animals were divided into four groups, *i.e.*; Control, ARDS, OES, and HI. Saline-lavage-induced lung injury was induced except for Control group. Thereafter, rabbits were ventilated with positive-end expiratory pressure (PEEP) at 10 cm H_2_O. The ARDS group received ventilation with the same PEEP without derecruitment. As intervention, OES and HI were performed in ARDS animals. OES was performed for 15 seconds at 150 mm Hg, whereas HI was performed with PEEP at 0 cm H_2_O and peak inspiratory pressure at +5 cm H_2_O for a minute. Total duration of the experiment was for 3 hours. OES and HI were performed every 15 minutes from beginning of the protocol.

**Results:**

PaO_2_ was maintained at about 400 mm Hg in both control and ARDS groups for the duration of this study, while in both OES and HI groups, PaO_2_ decreased continuously up to 3 hours, dropped to a mean (±SD) of 226 ± 28.9 and 97.0 ± 30.7 mmHg at 3 h, respectively. HI group had the lowest PaO_2_ in the present investigation. Histological lung injury score was the highest in HI group than other three groups. Pulmonary TNF-α and IL-8 levels were the highest in HI group compared to other groups, but without significant alterations at circulatory level in all the experimental groups.

**Conclusions:**

We show in the present study that hyperinflation following repeated OES deteriorate arterial oxygenation and the severity of lung injury in a rabbit model of ARDS undergoing mechanical ventilation.

## Background

Although mechanical ventilation is an indispensable basic life support tool and is essential in patients with acute respiratory distress syndrome (ARDS), special effort and care should be exercised to avoid causing ventilator associated lung injury (VALI). Because some have suggested that VALI is induced by cyclical overdistention and derecruitment of lung alveoli [1,2], the best practice in managing mechanical ventilation is ‘open lung and keep it open’ through high peak end-expiratory pressure (PEEP) [3].

Endotracheal suctioning is frequently needed in intubated and mechanically ventilated patients. Mechanically ventilated patients are not able to cough efficiently because of sedation and endotracheal tube. There are currently two methods for performing endotracheal suctioning, namely: open endotracheal suctioning (OES) and closed endotracheal suctioning (CES) [4,5]. In OES, disconnecting the patients from the mechanical ventilator [6,7] leads to severe hypoxemia and lung volume loss than is the case in CES [8-11]. However, more airway secretion is removed in OES than CES in both animal models and patients [9,12,13]. Based on these reasons, both techniques are sometimes used interchangeably [14].

Hyperinflation is one of the common techniques used by nurses and/or physiotherapists in patients receiving mechanical ventilation. It was originally called ‘bag squeezing’ because it is performed manually using a bag valve or a Jackson-Rees device. Hyperinflation generates a larger tidal volume than normal because it induces a higher inspiratory pressure [15] to stimulate a cough [16], recover oxygenation [17] and improve compliance [18], which is sometimes performed by combining with OES. A previous crossover study in mechanically ventilated patients showed that although hyperinflation performed before OES improved lung compliance and increased the volume of sputum, there was no significant difference noted in the extent of oxygenation between the OES study groups, with or without hyperinflation [16]. Previous studies have also indicated that hyperinflation poses risks of alveolar over-distention and instability to both intra thoracic pressure and cardiovascular hemodynamics [19-21]. In other words, although beneficial effects of performing hyperinflation have been documented, it is still unclear and inconclusive as to whether it (hyperinflation) is of any benefits to critically ill and mechanically ventilated patients, particularly to patients with ARDS [15].

We hypothesized that hyperinflation may induce VALI because of cyclical overdistention and derecruitment of lung alveoli if hyperinflation is performed inadequately. Thus, the aim of our current study was to assess the effects of repeated hyperinflation after repeated OES on lung injury based on morphological and molecular profiling, with subsequent alteration of arterial oxygenation in a saline lavage-induced surfactant depleted ARDS rabbit model.

## Methods

### Animal preparation

The study was performed in 30 male adult Japanese-White rabbits, weighing 2.5 to 3.5 kg. The current study protocol has been approved by the Ethics Committee of the Animal Resource Center of the University of Tsukuba. The rabbits were cared for in accordance with the guidelines of animal research ethics. Rabbits were anesthetized using sodium pentobarbital (75-150 mg, bolus infusion) and ketamine (10-25 mg/kg), and restrained in the supine position. A steady depth of anesthesia was maintained during the experiment by continuous infusion of sodium pentobarbital (5 mg/kg/h) and pancuronium bromide (0.1 mg/kg/h) via infusion pump through the ear vein. Normal saline (5 ml/kg/h) was continuously infused as maintenance fluid. After local infiltration of 1.0% lidocaine solution (0.25 mg/kg), a tracheostomy was performed and endotracheal tube (3.5 mm internal diameter) was inserted into the trachea. After that, the animals were ventilated using mechanical ventilator (LTV-1000 ventilator; Care Fusion, San Diego, CA), implementing pressure control mode, at the following ventilator settings: fraction of inspired oxygen (FIO_2_) 1.0, PEEP of 2 cm H_2_O, inspiratory time of 0.5 second. Inspiratory pressure and respiratory rate were adjusted to maintain constant expiratory tidal volume of 6 to 8 ml/kg to achieve normocarbia. Mechanical ventilation was continued in the same manner throughout the experiment, except for the adjustments of PEEP level described later.

Next, a catheter was inserted into the right carotid artery to sample blood for gas analysis (ABL 720; Radiometer Copenhagen, Copenhagen, Denmark) and to measure arterial blood pressure. Body temperature was monitored continuously by using a rectal probe and maintained between 38 and 39˚C using a heating pad.

After 30 min of stabilization of the above experimental condition, baseline data were recorded. The experimental animals were randomly assigned into four groups, namely; a) a healthy control (Control) with a 3 hour mechanical ventilation without ARDS induction and subsequent intervention (n = 6), b) ARDS only without undergoing intervention (ARDS, n = 8), c) ARDS with repeated OES (OES, n = 8) and d) ARDS + OES receiving hyperinflation intervention (HI, n = 8).

### Lung injury (ARDS) induction and subsequent interventions (OES and HI)

**Figure 1:**
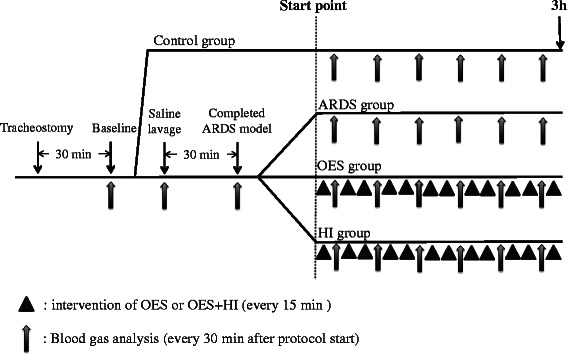
The sketch of the experimental protocol used in the present study. Same procedures were applied to 4 groups until baseline data were taken. After baseline data collection, rabbits of healthy control group received 3-hour mechanical ventilation without ARDS induction and subsequent intervention. Other groups (ARDS group, OES group and HI group) were applied each protocol; ARDS group was without undergoing intervention after saline lavage, OES group was performed the repeated open endotracheal suctioning every 30 min with ARDS, and HI group was added receiving hyperinflation intervention after open endotracheal suctioning (with ARDS).

After the rabbits were stabilized, lung injury was induced by a saline lavage protocol, with minor modification of the technique described previously by Lachmann et al [22]. The warmed sterile saline (18 ml/kg) was instilled via the endotracheal tube, and the rabbits were gently rocked from side to side in order to distribute saline uniformly. The animals were then shaken vigorously to facilitate the dispersion of normal saline and actively suctioned with a suction catheter. The lavage process was repeated until adequate lung injury was attained (defined as a PaO_2_ < 100 mm Hg) and each lavage was performed at 5 minute interval. After 30 minutes, blood gas sampling was done again. When PaO_2_ was maintained below 100 mm Hg, we assumed that generation of surfactant-depleted ARDS model was completed. After that, we elevated PEEP from 2 to 10 cm H_2_O, respectively, and the experimental protocol for subsequent intervention was begun. The inspiratory pressure limit was 25 cm H_2_O and the mandatory respiratory rate was consequently adjusted to maintain the PaCO_2_ in the range of 60–100 mm Hg, where possible, with a respiratory rate limited to 55/min. In the current study, OES and hyperinflation were applied as interventions to the generated ARDS model. The groups either received OES only or OES and hyperinflation. OES was performed every 15 minutes after the protocol was started. The endotracheal suctioning was applied for 15 seconds at a pressure of -150 cm H_2_O by using the 6 French suction catheter (Trachcare, Ballard Medical products. Draper, Utah). In the HI group, OES was immediately followed by hyperinflation using the following ventilator settings per minute; a) PEEP at 0 cm H_2_O and b) inspiratory pressure was set to add up the previous inspiratory pressure and 5 cm H_2_O as recruitment pressure. After the intervention protocol (OES and hyperinflation) was begun, data were collected every 30 minutes and whole blood samples were processed into serum and plasma hourly. Finally, at the end of a 3-hour experimental protocol, the rabbits were euthanized with bolus injection of sodium pentobarbital and lungs were prepared for histological and morphological analyses. The overview of the protocol is shown in Figure 1.

### Enzyme-linked immunosorbent assay

Left lung was removed and collected in cryotubes, snap frozen by immersion in liquid nitrogen and stored at -80°C. Enzyme-Linked Immunosorbent Assay (ELISA) was performed to measure the concentrations of tumor necrosis factor (TNF)-α, IL-1, Il-6 and Il-8 using rabbit specific commercial ELISA kit (IL-1; CUSABIO BIOTEC Co., Ltd, Wuhan, Hubei Province, P.R. China. TNF-alpha, IL-6 and IL-8; USCN Life Science & Technology, Missouri City, TX, USA).

### Histological analysis

The right lung was removed and inflated with 4% formaldehyde to a pressure of 20 cm H_2_O via trachea, and then fixed in 4% formaldehyde for over 24 hours, as previously described in already published articles [7,23-25]. Subsequently the lungs were divided into 4 sections and each section was stained with hematoxylin-eosin. Researchers (HK and MS) unaware and blinded to the nature and characteristics of the sample examined the hematoxylin-eosin stained slides microscopically using a quantitative evaluation system. Lung injury scores were made based on a thorough histopathological evaluation, including interstitial edematous alterations, inflammatory details, hyaline membrane injury/degeneration and the extent/severity of bronchiolar injury (0 = not present, 4 = severe and present throughout), as described previously [7,24]. Total lung injury score was calculated by summing each score obtained from the 4 evaluation categories, described above.

### Statistical analysis

We used the Kolmogorov-Smirnov test to determine whether normal distribution was obtained or not. All data presented in the Figures and Tables were expressed as mean ± SD, except the cytokine concentration data. Intergroup differences were compared by one-way ANOVA adjusting Bonferroni’s, and where time point data were available, the intergroup differences were analyzed by repeated-measures ANOVA with Bonferroni’s correction for multiple comparisons. Cytokine concentration was analyzed by Kruskal-Wallis test adjusting Steel-dwass, and expressed as median (interquartile range). All tests were performed using IBM-SPSS version 21.0 software (IBM-SPSS Inc., Chicago, IL, USA).

## Results

A total of eight rabbits were assigned to each treatment group, namely ARDS, OES, and HI, and six rabbits were assigned to the healthy control group.

### Baseline characteristics

Table 1 summarizes the baseline characteristics of the rabbits used in the current study. There was no significant difference among animals with respect to body weight, pH, PaO_2_, PaCO_2_, hemodynamic variables, lung mechanics prior to induction of lavage (ARDS) and total number of lavages.

**Table 1:** **The baseline characteristics**

	Control	ARDS	OES	HI	p value
Body weight (kg)	3.00 ± 0.12	3.04 ± 0.19	2.87 ± 0.10	2.92 ± 0.15	.128
The number of lavage (times)	—	4 ± 1	3 ± 1	3 ± 1	.934
pH	7.40 ± 0.07	7.41 ± 0.08	7.43 ± 0.07	7.47 ± 0.05	.213
PaCO_2_ (mm Hg)	44.4 ± 9.11	44.6 ± 4.72	39.7 ± 8.37	37.9 ± 5.88	.202
PaO_2_ (mm Hg)	413 ± 81.9	415 ± 41.2	449 ± 43.2	442 ± 40.4	.394
Lactate (mmol/l)	1.67 ± 0.96	0.96 ± 0.60	1.14 ± 0.68	1.58 ± 0.67	.266
HR (bpm)	297 ± 2.63	282 ± 35.6	251 ± 47.2	259 ± 32.8	.133
MAP (mm Hg)	126 ± 20.1	120 ± 16.0	109 ± 12.3	116 ± 13.2	.249
PIP (cm H_2_O)	12.7 ± 0.95	12.9 ± 0.35	13.6 ± 2.54	12.5 ± 1.31	.513
RR	23.0 ± 3.55	21.6 ± 1.51	24.1 ± 8.17	24.3 ± 4.33	.743

MAP, mean arterial blood pressure; HR, heart rate; PIP, peak inspiratory pressure; RR, respiratory rate.

All values were mean ± SD.

### Gas exchange

**Figure 2:**
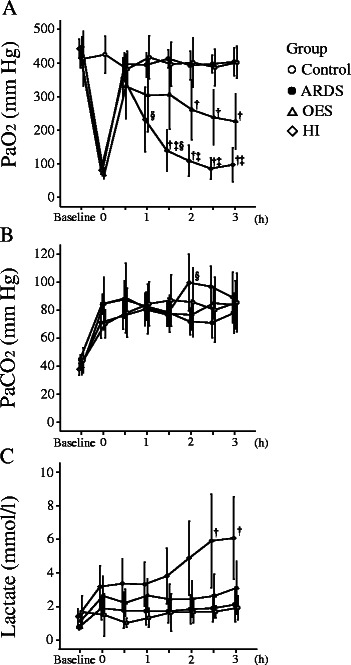
Sequential transition of blood gas analysis; **A)** PaO_2_, **B)** PaCO_2_ and **C)** Lactate in Control group (without lavage; *open circle*), ARDS group only (without OES and HI; *closed circle*); OES group (without HI; *open triangle*); and OES + HI group (*open rhombus). OES = open endotracheal suctioning; HI =* hyperinflation; ARDS = acute respiratory distress syndrome. ^†^*p* < 0.05 vs. Control group; ^‡^*p* < 0.05 vs. OES group; ^§^*p* < 0.05 vs. previous adjacent time point.

Figure 2 shows the time course profile of *A*) PaO_2_, *B*) PaCO_2_, and *C*) Lactate among four groups of the present investigation. After the induction of lung injury in all three groups with lavage (ARDS), PaO_2_ was reduced to a mean (±SD) of 53.2 ± 10.8 in ARDS group only, 56.6 ± 10.2 in OES group, and 49.9 ± 10.8 in HI group, respectively, compared to that of Control group, without lavage (*p* = .0001), indicating similarity in trend of PaO_2_ reduction in all groups of ARDS before further intervention (either OES or HI or both). After PEEP levels were increased to 10 cm H_2_O from the level of 2 cm H_2_O, PaO_2_ increased to over 300 mm Hg in all the study groups. In Control group without lavage and ARDS group without further intervention, PaO_2_ remained about 400 mm Hg during the rest of the study period. On the other hand, both in OES and HI groups, PaO_2_ decreased continuously and dropped to a mean of 226 ± 28.9 and 97.0 ± 30.7 mm Hg at 3 h, respectively. HI group demonstrated the highest level in reduction of PaO_2_ compared to that of OES group, thus clearly showing the significant deterioration of PaO_2_ in OES group after HI (*p* = .001). In contrast, PaCO_2_ did not show any significant difference at 3 h among all the study groups without a distinct time dependent change. In addition, HI failed to further up regulate the circulatory levels of lactate in OES group with a statistical significance, although blood lactate level significantly differed in between HI group and ARDS group with no intervention.

### Hemodynamic variables and respiratory mechanics

Table 2 shows the change of hemodynamic variables and respiratory mechanics in all the study groups over time. No significant difference was observed among all study groups, irrespective of time duration in regards to heart rate. Similar change was also observed when blood pressure alteration was taken into consideration. In addition, HI did not cause any significant change in pH level when performed in OES group compared to the OES group without HI. Regarding peak inspiratory pressure (PIP), in HI group it was higher than those in Control and ARDS groups at 3 h.

**Table 2:** **Sequential changes of hemodynamics and pulmonary parameters**

	Baseline	0	1	2	3
HR (bpm)					
Control	298 ± 23.5	270 ± 24.2	254 ± 23.8	240 ± 19.6	246 ± 24.9
ARDS	282 ± 16.6	240 ± 17.1	243 ± 16.8	264 ± 13.9	231 ± 17.6
OES	229 ± 15.6	213 ± 16.2	223 ± 15.9	234 ± 13.1	220 ± 16.6
HI	259 ± 16.6	207 ± 17.1	218 ± 16.8	216 ± 13.9	212 ± 17.6
MAP (mm Hg)					
Control	127 ± 7.40	106 ± 7.32	113 ± 7.32	105 ± 7.82	95.5 ± 11.8
ARDS	120 ± 5.23	102 ± 5.18	104 ± 5.45	98.4 ± 5.53	99.0 ± 8.33
OES	109 ± 4.94	102 ± 4.88	93.7 ± 5.56	103 ± 5.29	89.7 ± 7.85
HI	116 ± 5.23	113 ± 5.18	111 ± 5.45	98.3 ± 5.53	82.9 ± 8.33
pH					
Control	7.40 ± 0.04	7.26 ± 0.05	7.17 ± 0.05	7.19 ± 0.04	7.19 ± 0.03
ARDS	7.41 ± 0.03	7.13 ± 0.04^c^	7.13 ± 0.03	7.15 ± 0.03	7.12 ± 0.03
OES	7.43 ± 0.02	7.17 ± 0.03^c^	7.17 ± 0.03	7.15 ± 0.03	7.16 ± 0.02
HI	7.47 ± 0.03	7.19 ± 0.04^c^	7.15 ± 0.03	7.10 ± 0.03	7.08 ± 0.03
PIP (cm H_2_O)					
Control	12.8 ± 0.75	17.8 ± 1.44^c^	18.8 ± 0.97	20.0 ± 1.05	19.2 ± 1.01
ARDS	12.9 ± 0.53	20.6 ± 1.01^c^	21.3 ± 0.69	21.6 ± 0.74	20.4 ± 0.72
OES	13.9 ± 0.50	18.3 ± 0.96^c^	21.2 ± 0.65	21.4 ± 0.70	22.2 ± 0.67
HI	12.5 ± 0.53	19.6 ± 1.02^c^	21.0 ± 0.69	22.3 ± 0.74	23.7 ± 0.72^a,b^
RR					
Control	23.0 ± 2.66	33.0 ± 3.83^c^	34.5 ± 4.21	40.5 ± 4.66	39.7 ± 5.24
ARDS	21.6 ± 1.88	33.7 ± 2.71^c^	36.3 ± 2.98	35.0 ± 3.29	31.8 ± 3.71
OES	23.3 ± 1.88	36.3 ± 2.53^c^	38.0 ± 2.98	39.6 ± 3.29	40.5 ± 3.71
HI	24.3 ± 1.88	34.3 ± 2.53^c^	36.6 ± 2.98	39.5 ± 3.29	42.2 ± 3.71

MAP, mean arterial blood pressure; HR, heart rate; PIP, peak inspiratory pressure; RR, respiratory rate.

All values were mean ± SD.

^a^*p* < 0.05 vs. Control group; ^b^*p* < 0.05 vs. ARDS group; ^c^*p* < 0.05 vs. previous adjacent value in the same group.

### Lung morphology evaluation

**Figure 3:**
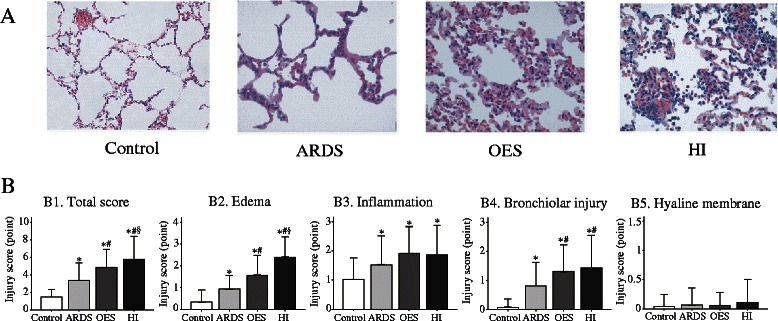
**(A)** Representative images of lung morphology from hematoxylin-eosin staining and **(B)** summary of quantitative analysis obtained from Control group (without lavage), ARDS group only (without OES and HI); OES group (without HI) and OES + HI group. *OES = open endotracheal suctioning; HI =* hyperinflation; ARDS = acute respiratory distress syndrome. The values are expressed as means ± SD. *p < 0.05 vs. Control group; ^#^*p* < 0.05 vs. ARDS group; ^§^*p* < 0.05 vs. OES group. Representative images were taken at magnification × 400.

Figure 3A shows the representative lung images taken from the experimental groups, with the summary of quantitative evaluation. Three notable changes in the current study for lung morphology evaluation are as follows; a) compared with Control group without lavage, all other three groups with lavage, irrespective of intervention, demonstrated a significant worsening in total lung injury score at 3 h of the study, implying that our current model of ARDS had significant lung injury (Figure 3B1); b) as consistent to other studies, OES caused further damage of lung morphology, as was evident from total lung injury score in ARDS injured lung (Figure 3B1); c) the unique notable finding is the observation of worsening or additional deterioration in lung injury of the OES group with HI as reflected in the total lung injury score (Figure 3B1). As evident from Figure 3B2, alteration in lung interstitial edema appears to be the primary contributor on total lung injury score exerted by HI. Changes in inflammation, bronchiolar injury, as well as hyaline membrane (Figure 3B3-B5), did not appear to cause any significant aggravation or had minimal contribution to total lung injury score after hyperinflation.

### Expression of cytokines

**Figure 4:**
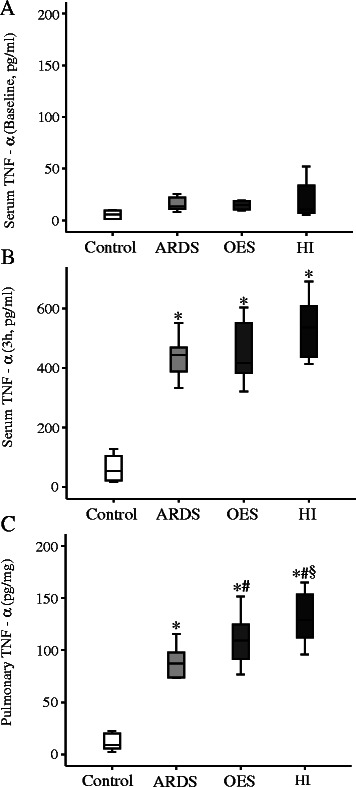
The expression of inflammatory cytokine TNF-α by ELISA; **A)** serum TNF-α (Baseline), **B)** serum TNF-α (3 h), **C)** pulmonary TNF-α (3 h) from Control group (without lavage), ARDS group only (without OES and HI); OES group (without HI) and OES + HI group. *OES = open endotracheal suctioning; HI =* hyperinflation; *ARDS* = acute respiratory distress syndrome. The end of boxes indicates the 25^th^ and 75^th^ percentiles, and the line in the bar indicates the median value. *p < 0.05 vs. Control group; ^#^*p* < 0.05 vs. ARDS group; ^§^*p* < 0.05 vs. OES group.

**Figure 5:**
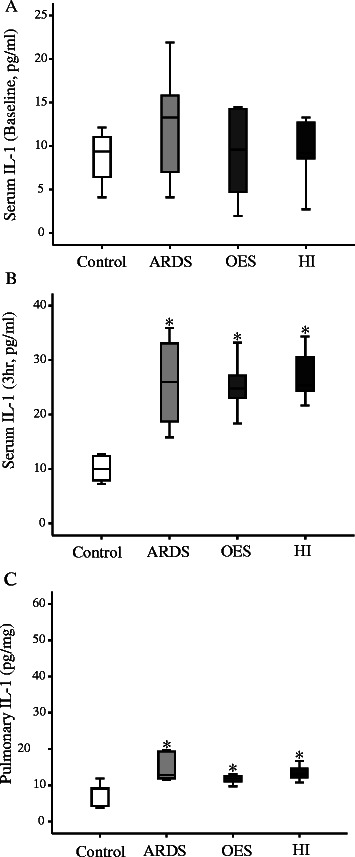
The expression of inflammatory cytokine IL-1 by ELISA; **A)** serum IL-1 (Baseline), **B)** serum IL-1 (3 h), **C)** pulmonary IL-1 (3 h) from Control group (without lavage), ARDS group only (without OES and HI); OES group (without HI) and OES + HI group. *OES = open endotracheal suctioning; HI =* hyperinflation; *ARDS* = acute respiratory distress syndrome. The end of boxes indicates the 25^th^ and 75^th^ percentiles, and the line in the bar indicates the median value. *p < 0.05 vs. Control group.

**Figure 6:**
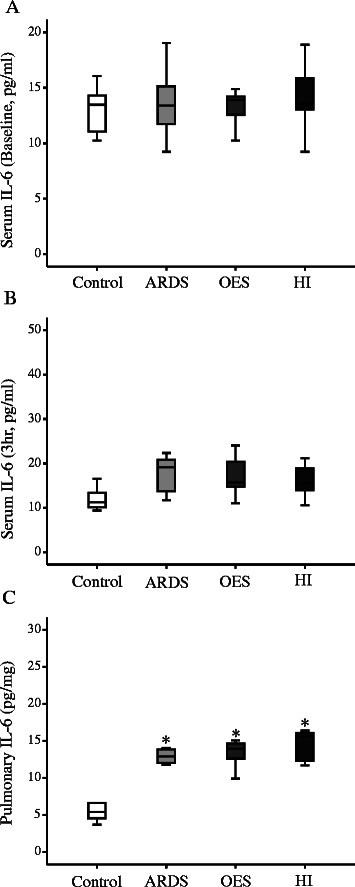
The expression of inflammatory cytokine IL-6 by ELISA; **A)** serum IL-6 (Baseline), **B)** serum IL-6 (3 h), **C)** pulmonary IL-6 (3 h) from Control group (without lavage), ARDS group only (without OES and HI); OES group (without HI) and OES + HI group. *OES = open endotracheal suctioning; HI =* hyperinflation; *ARDS* = acute respiratory distress syndrome. The end of boxes indicates the 25^th^ and 75^th^ percentiles, and the line in the bar indicates the median value. *p < 0.05 vs. Control group.

**Figure 7:**
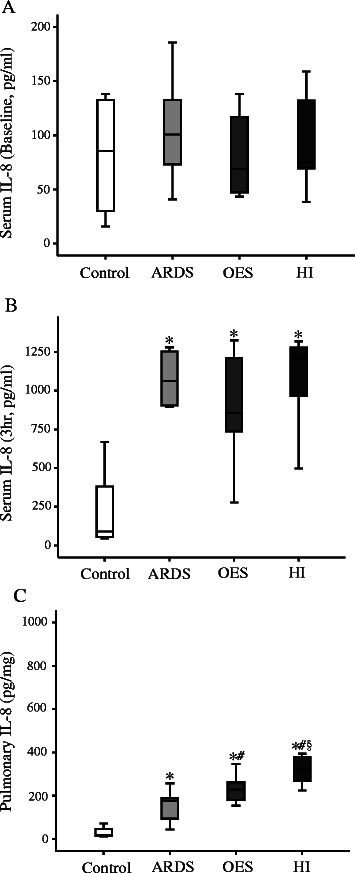
The expression of inflammatory cytokine IL-8 by ELISA; **A)** serum IL-8 (Baseline), **B)** serum IL-8 (3 h), **C)** pulmonary IL-8 (3 h) from Control group (without lavage), ARDS group only (without OES and HI); OES group (without HI) and OES + HI group. *OES = open endotracheal suctioning; HI =* hyperinflation; *ARDS* = acute respiratory distress syndrome. The end of boxes indicates the 25^th^ and 75^th^ percentiles, and the line in the bar indicates the median value. *p < 0.05 vs. Control group; ^#^*p* < 0.05 vs. ARDS group; ^§^*p* < 0.05 vs. OES group.

In the current study, there was no significant difference at the circulatory level for potential inflammatory cytokine like TNF-α among all study groups at 3 h (Figure 4B). On the other hand, pulmonary TNF-α levels were significantly elevated in all three groups with lavages (ARDS) compared to that of Control group without lavage. HI group had further elevated levels of pulmonary TNF-α compared to the OES group without hyperinflation (Figure 4C). Levels of both circulatory and pulmonary IL-1 and -8 were elevated with the induction of ARDS (Figures 5 and 6). Levels of pulmonary IL-8 in HI group was the highest in all three groups with lavages, while no significant difference was noted in the circulatory levels among the three groups. Also, no significant differences in IL-6 levels of both pulmonary and circulatory expression among the three groups with ARDS were noted (Figure 7).

## Discussion

This is the first study that explores the effects of hyperinflation on respiratory and hemodynamic parameters, lung morphology, as well as circulatory and pulmonary profile of inflammatory cytokine in a saline lavage-induced surfactant depleted rabbit model of ARDS with repeated OES undergoing mechanical ventilation. Thus, hyperinflation was found to worsen the respiratory parameter, lung morphology and expression of pulmonary TNF-α and IL-8 levels in the ARDS model undergoing mechanical ventilation with repeated OES.

Saline-lavage-induced lung injury model has been extensively investigated as an established model of ARDS [7,23,25,26], and the results of the present study are consistent with previous studies [7,23,26]. A previous review [27] suggested that it was relatively easier to use this model (cited here) for demonstrating ventilator-induced lung injury (VILI) when the effects of repeated endotracheal suctioning, as well as hyperinflation were studied. This is because the baseline injury in the lung following saline lavage is not associated with severe morphological impairment in this model. However, a study [28] highlights some of the drawbacks of the saline lavage model, *i.e.*, **1**) the pathology of ARDS is not reproducible in the saline lavage model; **2**) it is more recruitable than other models, **3**) and the model’s impaired oxygenation greatly depends on PEEP and inspiratory airway pressure. Indeed, the severity and extent of lung injury at the baseline level with saline lavage in the current model might be different pathophysiologically from those of other established ARDS models, such as those induced by injection of oleic acid, and intra-tracheal instillation of Escherichia coli [29]. Thus, the current study has some limitations arising from the use of the experimental ARDS model in as far as translating the current data to a clinical setting.

Regarding the OES protocol in the present study, we strictly followed the American Association for Respiratory Care (AARC) guidelines [4]. Under such suctioning protocol, the present study was able to demonstrate the significant drop of arterial oxygenation after OES in ARDS model, as already shown in previous studies [7,23-26]. This fact implies that the stability and reproducibility of both experimental models, as well as the suctioning protocol in the current experimental protocol as consistent to previous studies. The most notable finding in the present study is the demonstration of further deterioration of arterial oxygenation by hyperinflation in ARDS animals undergoing mechanical ventilator support with repeated OES. Although there was a gradual reduction of oxygenation in OES with hyperinflation over time, a relatively sharp drop was observed in the early period of the experiment, notably between 1.5 h and 2 h. Such a pattern in reduction of arterial oxygenation by hyperinflation as revealed by the current study for now cannot be compared with other studies, as this is the first study using such an experimental design.

Although we do not know for now the exact mechanism that may mediate the additional drop in arterial oxygenation induced by hyperinflation in combination with OES, the higher peak inspiratory pressure in HI group at 3 h may suggest decreased lung compliance, which might be one of the contributing factors to the exacerbation of arterial oxygenation reduction. In addition, the severity of lung injury in ARDS with hyperinflation may also help explain further the hyperinflation-induced deterioration of arterial oxygenation in the current study.

In the histological analysis, hyperinflation deteriorated lung injury induced by OES, as was evident from the total injury score. Aggravation of lung edema by hyperinflation may have led to a decrease in lung compliance, as well as aeration of lung alveoli. Bronchiolar injury score was significantly elevated in three ARDS groups compared with Control group, and OES exacerbated the lung injury, as already demonstrated in the previous study [30]. Regarding the scoring derived from the hyaline membrane injury/degeneration, there was no significant difference among the four groups of current experimental setting, implying that the duration used in the current study is inadequate to cause any detrimental effect on the lung hyaline membrane formation.

In a number of studies, levels of TNF-alpha and IL-8 have been shown to be altered in ARDS models/subjects [23,31,32]. Accordingly, we found elevated expression of both TNA-alpha and IL-8 in the lung tissues of the present ARDS models. One of the crucial findings of the present study is that HI induced further expression of both pulmonary TNF-α and IL-8 levels in our experimental ARDS rabbit model. In contrast, in the present study, circulatory and pulmonary IL-1 levels were not found to be significantly different among the three groups with ARDS. Also, in our current results, IL-6 levels were not aggravated by hyperinflation in ARDS lung tissues like that of IL-1. Thus, from the current investigation of cytokine profile, hyperinflation only upregulates levels of pulmonary TNF-α and IL-8.

Based on the present data, here, we consider four possible mechanisms that were induced deterioration of oxygenation, histological lung injury, and cytokine release; namely barotrauma, volutrauma, atelectrauma and biotrauma. In our speculation, this locally up regulated pulmonary TNF-α and IL-8 levels induced by hyperinflation may potentially contribute to the development of biotrauma, one of the crucial components of ventilator-induced lung injury in the ARDS subjects undergoing mechanical ventilation [33,34]. If categorized sequentially or chronologically, based on their ability to cause lung injury, atelectrauma would be the first because of repetitive alveolar recruitment and derecruitment induced by the release of PEEP during hyperinflation [35]. The previous study showed that mechanical and cyclic stretch induced the expression of transcription factors related to the expression induction of interleukins through the signaling pathways of p38 and NF-κB [36]. If this previous result is applied to our current findings, hyperinflation may act as the cyclic stretch to stimulate further release of cytokines. Although we did not have any direct evidence regarding the occurrence of atelectrauma and/or volutrauma in the present study, we consider the possibility that hyperinflation performed in the present study may lead to atelectrauma and/or volutrauma. The previous studies showed that respiratory rate is very important in determining the magnitude of PaO_2_ oscillation [28], and that it is possible that cyclic recruitment is strongly related to atelectrauma [37]. The fact that a sharp drop in PaO_2_ was observed in the early period of our experiment (between 1.5 h and 2 h) in HI group, this observed drop in PaO_2_ may be due to the hyperinflation-mediated cyclic recruitment and derecruitment. Thus, to clarify these mechanisms and relationship, further study is necessary to determine the exact lung volume with specific device system and technology.

The current study has several potential limitations; 1) firstly, a surfactant-depleted ARDS rabbit model may not reflect conditions in human and saline lavage model itself has some limitations, as mentioned above; 2) secondly, several crucial respiratory parameters were lacking, notably, measured PEEP value, inspiration and expiration time, dynamic lung compliance, and the venous admixture (Qs/Qt); 3) thirdly, we were unable to measure the exact lung volume using electrical impedance tomography and/or computed tomography. Such assessment of lung volume might have explored the mechanism of the lung injury induced by HI; 4) fourthly, since the clinical use of hyperinflation in Japan may not be universal, the current study design is not necessarily the global standard; 5) further, the frequency of hyperinflation used in the current study design may not match that used in clinical settings, and it is different from the clinical setting because it is an animal study; 6) also, in order to have a clearer picture of the cytokine profile using the current study design, longer duration of OES and hyperinflation protocol may be necessary; 7) lastly, lung histological assessment and scoring should be performed under more reliable conditions, such as decreased levels of PEEP compared to that of the present study and non-edematous condition. Finally, as evident from the present study and also from other investigations, we concluded that repeated OES and hyperinflation under high PEEP deteriorates gas exchange. Further, the efficacy of repeated hyperinflation as a method/technology to recover oxygenation after OES is questioned and the necessity of performing hyperinflation should be re-assessed, re-visited and re-investigated. In addition, we consider that the potential mechanisms underlying the deterioration of arterial oxygenation and lung injury following hyperinflation in ARDS model should be investigated in depth in future studies.

## Conclusions

Hyperinflation following repeated OES can deteriorate arterial oxygenation and lung injury in a rabbit model of saline lavage induced lung injury undergoing mechanical ventilation. Further studies are needed to elucidate the effects of hyperinflation following repeated OES.

## Abbreviations

ARDS: Acute respiratory distress syndrome
CES: Closed endotracheal suctioning
HI: Hyperinflation
OES: Open endotracheal suctioning
PEEP: Peak end expiratory pressure
PIP: Peak inspiratory pressure
RM: Recruitment maneuver
TNF-α: Tumor necrosis factor-alpha
VILI: Ventilator induced lung injury
